# Cigarette Smoke-Related Hydroquinone Dysregulates MCP-1, VEGF and PEDF Expression in Retinal Pigment Epithelium in Vitro and in Vivo

**DOI:** 10.1371/journal.pone.0016722

**Published:** 2011-02-28

**Authors:** Marianne Pons, Maria E. Marin-Castaño

**Affiliations:** Department of Ophthalmology, Miller School of Medicine, Bascom Palmer Eye Institute, University of Miami, Miami, Florida, United States of America; Istituto Dermopatico dell'Immacolata, Italy

## Abstract

**Background:**

Age-related macular degeneration (AMD) is the leading cause of legal blindness in the elderly population. Debris (termed drusen) below the retinal pigment epithelium (RPE) have been recognized as a risk factor for dry AMD and its progression to wet AMD, which is characterized by choroidal neovascularization (CNV). The underlying mechanism of how drusen might elicit CNV remains undefined. Cigarette smoking, oxidative damage to the RPE and inflammation are postulated to be involved in the pathophysiology of the disease. To better understand the cellular mechanism(s) linking oxidative stress and inflammation to AMD, we examined the expression of pro-inflammatory monocyte chemoattractant protein-1 (MCP-1), pro-angiogenic vascular endothelial growth factor (VEGF) and anti-angiogenic pigment epithelial derived factor (PEDF) in RPE from smoker patients with AMD. We also evaluated the effects of hydroquinone (HQ), a major pro-oxidant in cigarette smoke on MCP-1, VEGF and PEDF expression in cultured ARPE-19 cells and RPE/choroids from C57BL/6 mice.

**Principal Findings:**

MCP-1, VEGF and PEDF expression was examined by real-time PCR, Western blot, and ELISA. Low levels of MCP-1 protein were detected in RPE from AMD smoker patients relative to controls. Both MCP-1 mRNA and protein were downregulated in ARPE-19 cells and RPE/choroids from C57BL/6 mice after 5 days and 3 weeks of exposure to HQ-induced oxidative injury. VEGF protein expression was increased and PEDF protein expression was decreased in RPE from smoker patients with AMD versus controls resulting in increased VEGF/PEDF ratio. Treatment with HQ for 5 days and 3 weeks increased the VEGF/PEDF ratio in vitro and in vivo.

**Conclusion:**

We propose that impaired RPE-derived MCP-1-mediated scavenging macrophages recruitment and phagocytosis might lead to incomplete clearance of proinflammatory debris and infiltration of proangiogenic macrophages which along with increased VEGF/PEDF ratio favoring angiogenesis might promote drusen accumulation and progression to CNV in smoker patients with dry AMD.

## Introduction

Age-related macular degeneration (AMD) is the main cause of untreatable blindness among older adults in the developed world and has a devastating impact on quality of life [1], [2], [3], [4]. Although much effort is invested in understanding this condition, there is neither a cure nor a way to prevent it, and treatment options are very limited. AMD affects more than 1.75 million individuals in the United States [5] and it is estimated that more than 300,000 new cases are diagnosed annually [1], [3]. Unless better preventive treatments emerge, this number is expected to climb threatening to reach epidemic proportions given the longer life expectancies [6]. The development of novel therapeutic approaches to provide more effective disease control is critical and requires a better understanding of the different pathophysiological processes underlying the critical steps in the etiology of AMD.

Histopathological signs of dry AMD, the early stage of the disease, include the presence of waste material in the form of focal deposits named drusen below the retinal pigment epithelium (RPE) and within Bruch's membrane, a stratified extracellular matrix situated between the choriocapillaris and RPE [4], [7]. Drusen increase in size and number as the disease progresses. Geographic atrophy represents the advanced stage of dry AMD and is characterized by progressive destruction of RPE cells and photoreceptors. Only a fraction of patients with drusen will develop wet AMD, the most rapidly progressing form which accounts for 80 to 90% of cases of severe vision loss related to the disease. The presence, size and area covered by drusen confer a significant risk for the development and progression to wet AMD [8], [9], [10]. Wet AMD is characterized by the growth of leaky new abnormal blood vessels under the RPE from the subjacent choroid known as choroidal neovascularization (CNV). Our understanding of the two forms of AMD has increased substantially, yet there is still much debate as to why and how the disease progresses and what sequence of cellular events lead to the progression of dry to wet AMD. Overall, the mechanism by which drusen might elicit CNV remains undefined.

Although the pathophysiology of AMD is not yet fully understood, this multifactorial degenerative disease likely arises from a complex interaction of genetic and environmental risk factors [2], [11] among which cigarette smoking is the single most important for onset and severity of all forms of AMD [4], [12], [13], [14], [15]. Overwhelming evidence shows that smokers have a greater prevalence of AMD compared with non smokers [13], [14], [16], [17], [18], [19], [20], [21]. Former smokers remain at high risk for AMD [19] and passive smoking (also called secondhand smoke or environmental tobacco smoke) almost doubles the risk of AMD [22]. Cigarette smoking may contribute to the etiology of AMD by causing oxidative damage to the RPE strategically located between the neural retina and the vascular choroid [23]. Tar within cigarette smoke contains a large number of pro-oxidant compounds among which hydroquinone (HQ) is the most abundant. HQ is an oxidant of special relevance due to its presence not only in cigarette smoke but also in foodstuff and atmospheric pollutants as well as its widespread occurrence in nature. We and others have shown that smoking and HQ cause oxidative damage to the RPE in vitro and vivo which might play a key role in the pathogenesis of AMD [23], [24], [25], [26], [27], [28], [29]. However, little is known about how the RPE may contribute to the progression of dry AMD to the wet form of the disease and the role played by cigarette smoke-related HQ-induced oxidative stress.

Aberrant expression of chemokines occurs in a variety of diseases that have an inflammatory component. A growing collection of evidence suggests that inflammation is a key cellular process that plays a central role in the pathogenesis of AMD [30], [31], [32], [33] and its progression to CNV [34]. Highly specialized RPE cells play a pivotal role in the maintenance of the outer retina by secreting several cytokines including monocyte chemoattractant protein-1 (MCP-1) [35], [36], which has been suggested to be implicated in the pathogenesis of AMD [37], [38]. RPE cells can secrete MCP-1 in the direction of choroidal blood vessels during inflammatory responses therefore suggesting that RPE cells might promote macrophage recruitment to the choroid from circulating monocytes [39]. MCP-1 expression has not been investigated in RPE from patients with AMD nor has been the regulation of RPE-derived MCP-1 expression following cigarette smoke-related HQ-mediated oxidative injury.

Angiogenesis is a highly complex biological process that involves a delicate balance between numerous stimulators and inhibitors, each regulated by multiple control systems. CNV-related angiogenesis requires an alteration in the concentration of molecules that stimulate or inhibit growth of new blood vessels [40]. Vascular endothelial growth factor (VEGF), constitutively produced and secreted by RPE in culture [41] [42], [43], [44], [45], [46], , is a major angiogenic cytokine central to the development of wet AMD [42], [43], [44], [45]. VEGF regulates endothelial cells proliferation, migration and survival [50]. Interestingly, secretion of VEGF by RPE cells is polarized towards Bruch's membrane [51]. There is ample clinical evidence that VEGF expression is increased in surgically excised AMD-associated choroidal neovascular membranes [46], [47], [48]. Eyes with early forms of AMD have increased expression of VEGF in the RPE and the vitreous of eyes with CNV have increased concentration of VEGF [46]. Similar observations have been made in animal models of CNV [43], [52]. Furthermore, Reich et al reported that subretinal injection of VEGF siRNA significantly inhibited the growth of laser-induced CNV in a mouse model [49]. PEDF, a potent angiogenic inhibitor [53] secreted by RPE cells [54], [55], [56], counterbalances the effects of VEGF and modulates the formation of CNV [54], [55]. A decrease in PEDF expression has been reported in eyes with AMD, therefore disrupting the critical balance between VEGF and PEDF that may lead to pathological angiogenesis and be permissive for the development of CNV [54]. PEDF levels decline in the vitreous of patients with CNV [56]. However, until now, there is no report in the literature examining VEGF and PEDF expression in RPE from AMD patients or evaluating whether or not cigarette smoke-related HQ-induced oxidative stress has the potential to dysregulate the VEGF/PEDF balance in RPE cells.

Given their critical role in AMD and that oxidative damage to the RPE and inflammation appear to be central in the pathogenesis of the disease, we studied the effect of different HQ concentrations and durations of exposure on the regulation of MCP-1, VEGF and PEDF expression in RPE in vitro and in vivo. We also studied the expression of these chemokines in RPE from smoker patients diagnosed with AMD. Better understanding of the cellular events leading to wet AMD is critical to the development of new therapeutic approaches that may help slow down the progression of this vision threatening disease in the elderly.

## Results

### MCP-1 expression is downregulated in RPE from smoker patients with AMD

**Figure 1:**
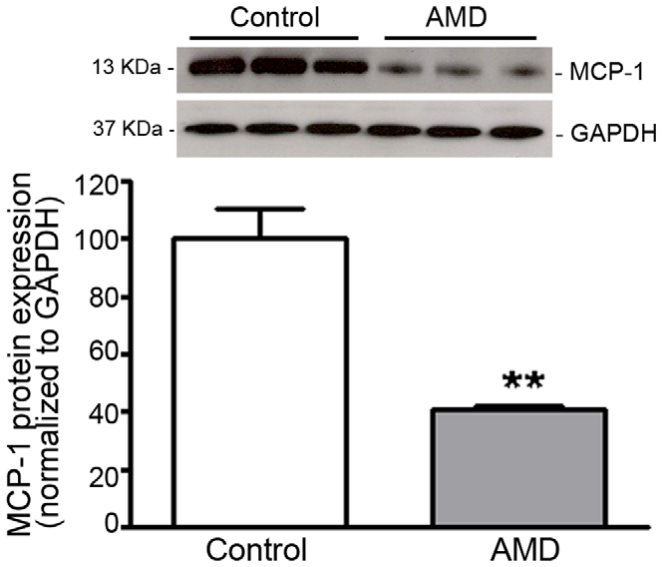
MCP-1 expression is decreased in RPE from smoker AMD patients. MCP-1 protein expression was evaluated by Western blot in RPE lysates from 3 smoker donors with AMD and 3 age-matched non smoker controls with no known history of eye disease. GAPDH served as loading control. *Top:* representative Western blots of the indicated proteins. The numbers to the left are molecular weights in kilodaltons (KDa). *Bottom*: average densitometry results. Data are expressed as percentage of control and are means ± SE. **p<0.01 versus control.

Declining production of MCP-1 by RPE and macrophage recruitment may be implicated in AMD [37]. MCP-1 expression has not been studied in human RPE from smoker AMD patients. With the use of Western blot analysis, we investigated the expression of MCP-1 in RPE lysates from smoker patients diagnosed with AMD and control non-smoker donors with no known history of eye diseases. In normal donors, we found that RPE express high levels of MCP-1 (Figure 1). For the first time, we showed that MCP-1 expression is robustly downregulated by ∼59% in RPE from AMD patients compared with non-smoker controls (41.2±0.8 versus 100.0±10.2%, p<0.01) (Figure 1).These findings suggest that MCP-1 might play an important role in the pathobiology of AMD. Diminished expression of MCP-1 might have implications in the recruitment of scavenging macrophages which are responsible for clearing debris from the sub-RPE space.

### Sustained and repetitive oxidative injury with HQ decreases MCP-1 expression in RPE cells

**Figure 2:**
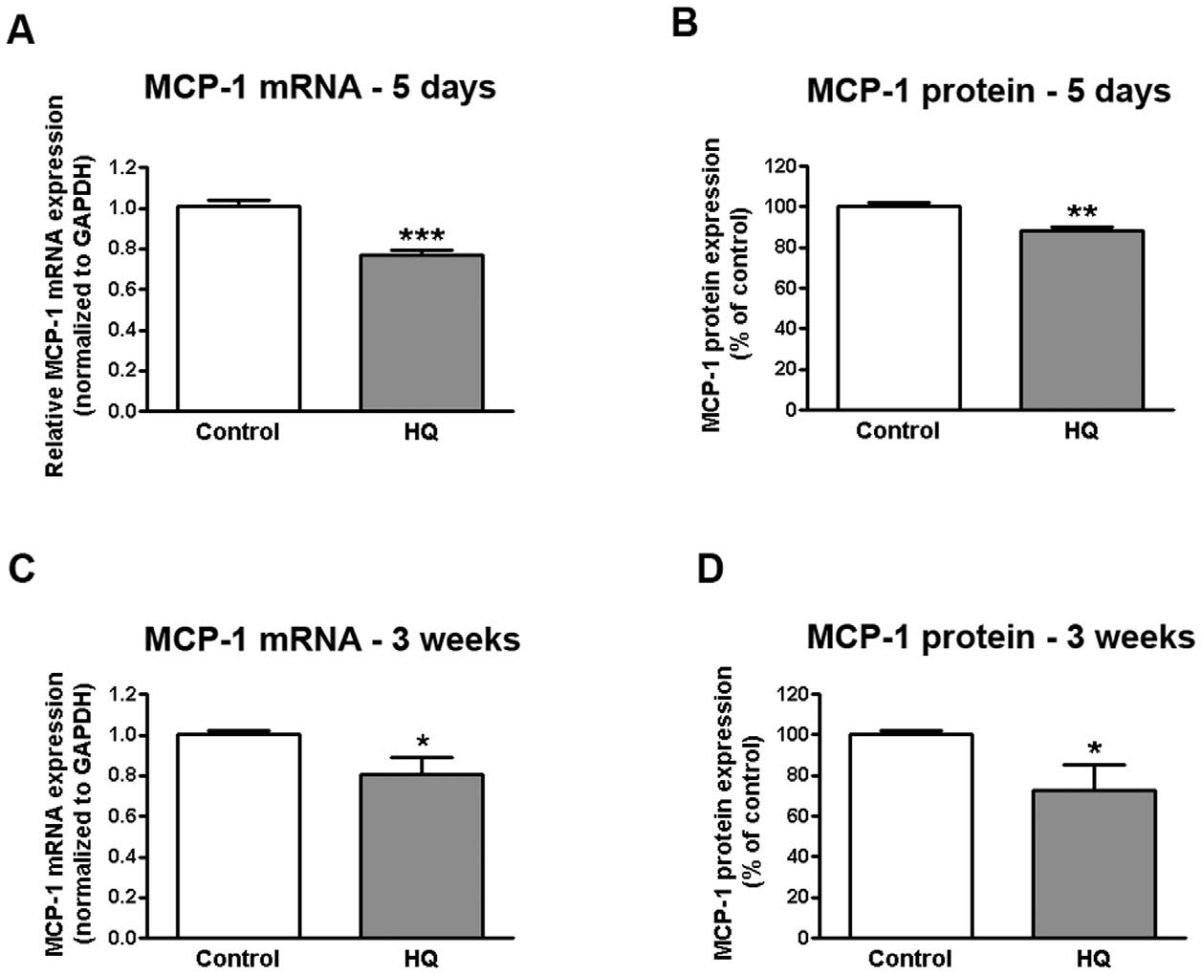
Sustained and repetitive HQ-induced oxidative injury decreases on MCP-1 expression in human RPE cells. Confluent serum-starved ARPE-19 cells were treated with (**A, B**) 10 µM HQ every 24 hours for 5 consecutive days or (**C, D**) 50 µM HQ every 4 days for 24 hours for 3 consecutive weeks in phenol red-free 0.1% FBS medium. Total RNA was extracted to assess MCP-1 mRNA expression by real-time PCR (**A, C**). GAPDH was used as internal control. Supernatants were collected to assess MCP-1 protein concentration by ELISA (B, D). Data are mean± SE and represent the average results of 3 independent experiments run in duplicate. * is p<0.05, ** is p<0.01 and *** is p<0.001 versus control.

Prolonged oxidative injury has been suggested as one of the causes of a number of retinal pathologic conditions, including AMD. We have previously investigated the effects of repetitive acute (6 hours every 3 days for 4 weeks) and transient (6 hours followed by a recovery phase, every 5 days for 6 weeks) exposure of ARPE-19 cells to HQ on matrix metalloproteinase-2 activity and extracellular matrix turnover relevant to the pathogenesis of dry AMD [26]. Regulation of RPE-derived MCP-1 expression following HQ-mediated oxidative injury has not been investigated. Here, we found that sustained exposure of ARPE-19 cells to HQ 10 µM every 24 hours for 5 consecutive days decreased MCP-1 mRNA expression by 24% compared with control cells (0.76±0.02 versus 1.0±0.03, p<0.001) (Figure 2A) with a concomitant moderate ∼12% decrease in MCP-1 protein released in the supernatants as measured by ELISA (88.27±1.8 versus 100.0±2.1%, p<0.01) (Figure 2B) without causing any cell death (94.6±6.1% of cells survived after treatment with NT versus 100.0±3.5% in control conditions, p>0.05). We also tested the effect of repetitive long-term exposure to sub-lethal oxidative stress by treating ARPE-19 cells with HQ 50 µM every 4 days for 24 hours for 3 consecutive weeks. We observed a ∼20% decline in MCP-1 mRNA expression (0.80±0.9 versus 1.0±0.01, p<0.05) (Figure 2C) with a concomitant ∼27% decrease in protein secretion (72.95±12.27 versus 100.0±2.0% p<0.05) (Figure 2D) relative to control cells without any cell death (95.4±5.9% of cells survived after treatment with NT versus 100.0±1.3% in control conditions, p>0.05). These observations suggest that cigarette smoke-related HQ-induced downregulation of RPE-derived MCP-1 expression may play an important role in the pathogenesis of AMD.

### HQ-induced oxidative injury decreases MCP-1 expression in RPE/choroids from C57BL/6 mice

**Figure 3:**
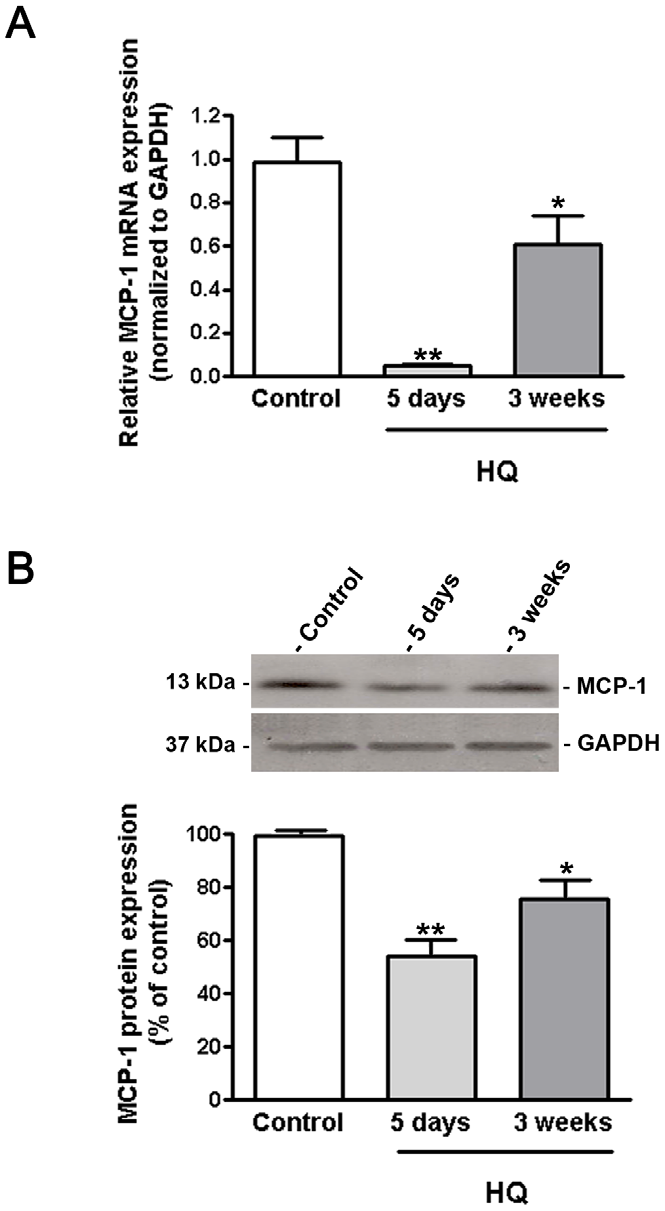
MCP-1 expression is decreased in RPE/choroids from mice exposed to HQ. (**A**) MCP-1 mRNA expression was downregulated in response to HQ-induced oxidative injury. Total RNA was extracted from microdissected RPE/choroid complexes after 5 days and 3 weeks of exposure to HQ in drinking water (0.8%). MCP-1 mRNA expression was measured by real-time PCR. GAPDH was used as internal control (n = 5 eyes per group). (**B**) MCP-1 protein expression was downregulated in response to HQ-induced oxidative injury. Total protein was extracted from microdissected RPE/choroid complexes after 5 days and 3 weeks of exposure to HQ in drinking water (0.8%). Equivalent amounts of protein from 5 eyes per group were pooled for each lane. MCP-1 protein expression was evaluated by Western blot and normalized to GAPDH. *Top:* representative Western blot gel. The numbers to the left are molecular weights in kilodaltons (KDa). *Bottom*: average densitometry results. Data are expressed as percentage of control and are means ± SE. * is p<0.05 and **p<0.01 versus control.

Based on the above observations obtained in vitro on ARPE-19 cells, we hypothesized that pro-oxidant HQ might also regulate MCP-1 expression in mice treated with 0.8% HQ in drinking water. A longer exposure for 5 days almost completely abolished MCP-1 mRNA expression compared with control mice (0.04±0.006 versus 1.0±0.12, p<0.001) (Figure 3A) but the expressed protein was only ∼46% downregulated (54.1±6.2 versus 100.0±0.01%, p<0.01) (Figure 3B). After 3 consecutive weeks of exposure to HQ, MCP-1 mRNA expression in RPE/choroids was dramatically downregulated compared with a 5 day-treatment but remained 39% lower compared with control mice (0.61±0.13 versus 1.0±0.12, p<0.05) (Figure 3A). At 3 weeks, MCP-1 expression was decreased by ∼30% at the translational level (70.5±4.3 versus 100.0±0.01%, p<0.01) (Figure 3B). These observations are consistent with aforementioned data on ARPE-19 cells at 5 days and 3 weeks indicating that cumulative cigarette-smoke related HQ-induced oxidative injury might decline RPE-derived MCP-1expression therefore inhibiting the recruitment of scavenging macrophages therefore playing a crucial role in the progression of AMD.

### VEGF-to-PEDF ratio is increased in RPE from smoker patients with AMD

**Figure 4:**
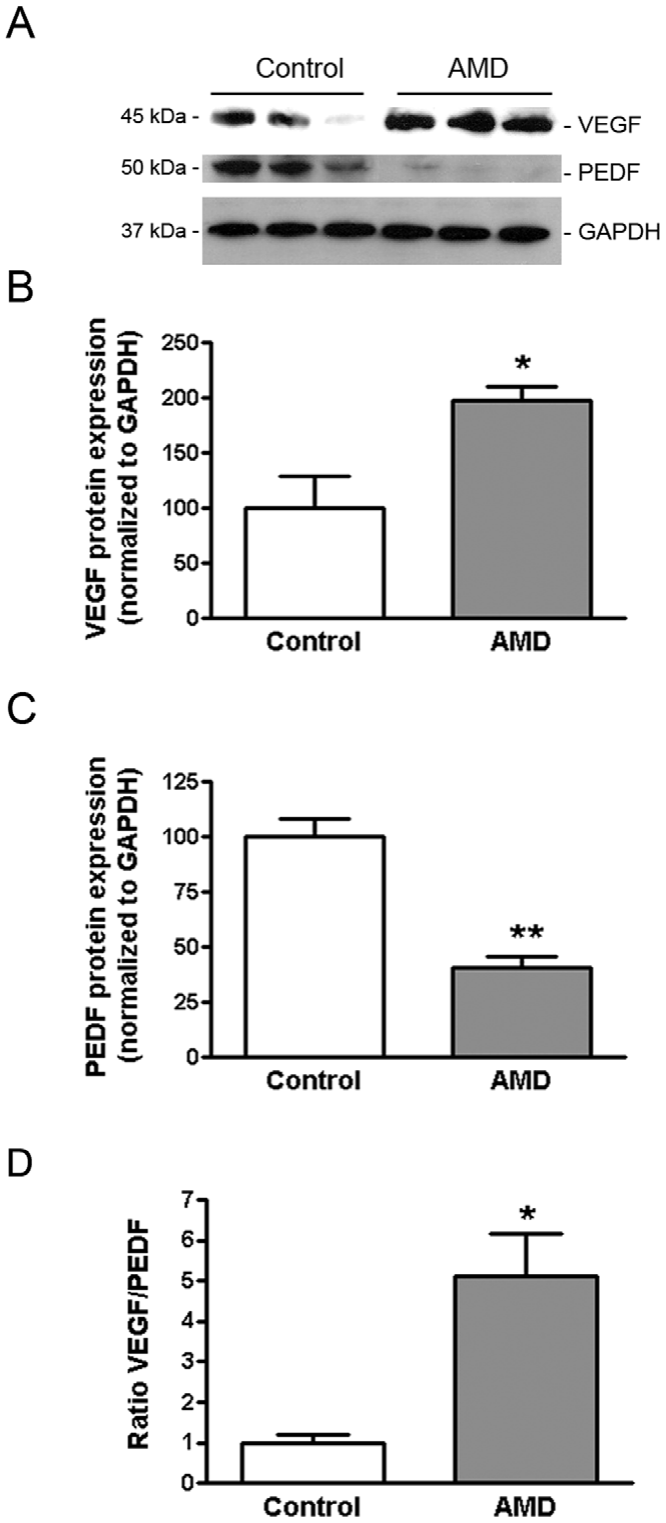
VEGF expression is increased and PEDF expression decreased in RPE from AMD patients. VEGF and PEDF protein expression was evaluated by Western blot in RPE lysates from 3 smoker donors with AMD and 3 non smoker controls with no known history of eye disease. GAPDH served as loading control. (**A**) Representative Western blots of the indicated proteins. The numbers to the left are molecular weights in kilodaltons (KDa). (**B, C**) Average densitometry results. (D) VEGF-to-PEDF protein ratio. Data are expressed as percentage of control and are means ± SE. * is p<0.05 and **p<0.01 versus control.

Dysregulated expression of VEGF and PEDF by RPE cells may be involved in the pathogenesis of CNV. Here, we reported that VEGF is expressed at higher levels (198.0±12.5 versus 100.0±28.9%, p<0.05) (Fig. 4A and 4B) and PEDF at lower levels (40.70±5.1 versus 100.0±7.9, p<0.01) (Fig. 4A and 4C) in RPE from AMD smoker patients compared to RPE from non-smoker control donors as shown by Western blot. Overall, the VEGF-to-PEDF ratio was increased by ∼4.0-fold (5.12±1.3 versus 100.0±0.2, p<0.05) (Fig. 4D) in RPE from smoker AMD patients relative to non-smoker control donors. Unbalanced expression of VEGF and PEDF might promote angiogenesis and therefore lead to the conversion of dry AMD to CNV.

### Sustained and repetitive oxidative injury with HQ increases the VEGF-to-PEDF ratio in RPE cells

**Figure 5:**
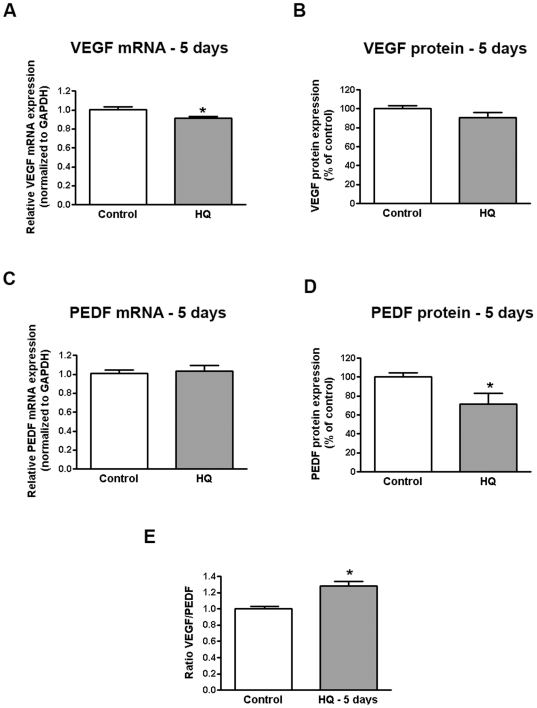
Increased VEGF-to-PEDF ratio in human RPE cells following sustained oxidative injury with HQ. Confluent serum-starved ARPE-19 cells were treated with 10 µM HQ every 24 hours for 5 consecutive days in phenol red-free 0.1% FBS medium. Total RNA was extracted to assess (**A**) VEGF and (**D**) PEDF mRNA expression by real-time PCR. GAPDH was used as internal control. Supernatants were collected to assess (**B**) VEGF and (**D**) PEDF protein concentration by ELISA. (**E**) VEGF-to-PEDF protein ratio. Data are mean± SE and represent the average results of 3 independent experiments run in duplicate. * is p<0.05 versus control.

We showed that VEGF mRNA expression was slightly decreased by ∼9% (0.91±0.01 versus 1.00±0.02, p<0.05) (Figure 5A) while VEGF protein expression remained unchanged (91.9±5.3 versus 100.0±3.2%, p = 0.17) in response to sustained exposure to HQ 10 µM every 24 hours for 5 consecutive days (Figure 5B). In addition, PEDF mRNA expression was not affected (Figure 5C) while the amount of PEDF protein released in the supernatants was decreased by ∼28% relative to control cells (71.47±11.34 versus 100.0±4.45, p<0.05) (Figure 5D). These results indicate a moderate ∼30% increase in VEGF/PEDF ratio (1.30±0.3 versus 1.00±0.01, p<0.05) which may switch the balance in favor of angiogenesis stimulation over inhibition (Fig. 5E).

**Figure 6:**
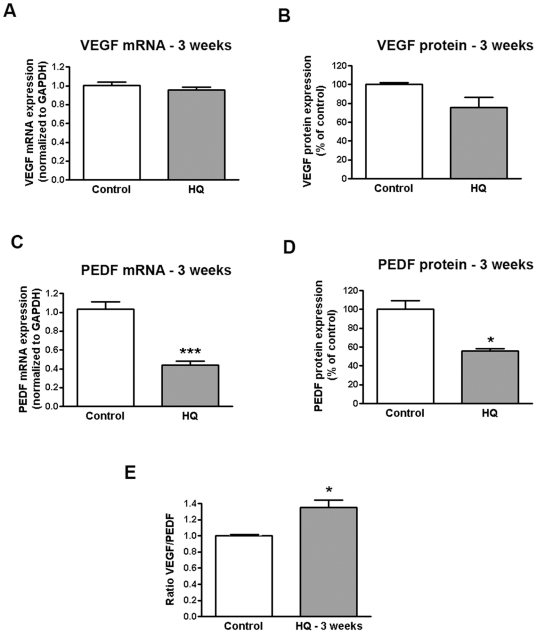
Increased VEGF-to-PEDF ratio in human RPE cells following repetitive oxidative injury with HQ. Confluent serum-starved ARPE-19 cells were treated with 50 µM HQ every 4 days for 24 hours for 3 consecutive weeks in phenol red-free 0.1% FBS medium. Total RNA was extracted to assess (**A**) VEGF and (**D**) PEDF mRNA expression by real-time PCR. GAPDH was used as internal control. Supernatants were collected to assess (**B**) VEGF and (**D**) PEDF protein concentration by ELISA. (**E**) VEGF-to-PEDF protein ratio. Data are mean± SE and represent the average results of 3 independent experiments run in duplicate. * is p<0.05 and *** is p<0.0001 versus control.

Following repetitive oxidative injury with HQ 50 µM every 4 days for 24 hours for 3 consecutive weeks, VEGF expression was not changed either at the transcriptional (Figure 6A) or the translational level (Figure 6B). Contrastingly, PEDF mRNA expression was robustly decreased by 60% (0.4±0.04 versus 1.0±0.08, p<0.0001) (Figure 6C) compared with untreated cells which translated into ∼46% decreased PEDF protein secretion in the supernatant (55.95±2.63 versus 100.0±9.59% p<0.01) (Figure 6D). These data demonstrate ∼35% increase in VEGF to PEDF ratio which may switch the balance in favor of angiogenesis stimulation over inhibition (Fig. 6E).

### HQ-induced oxidative injury dysregulates the balance between VEGF and PEDF expression in RPE/choroids from C57BL/6 mice

**Figure 7:**
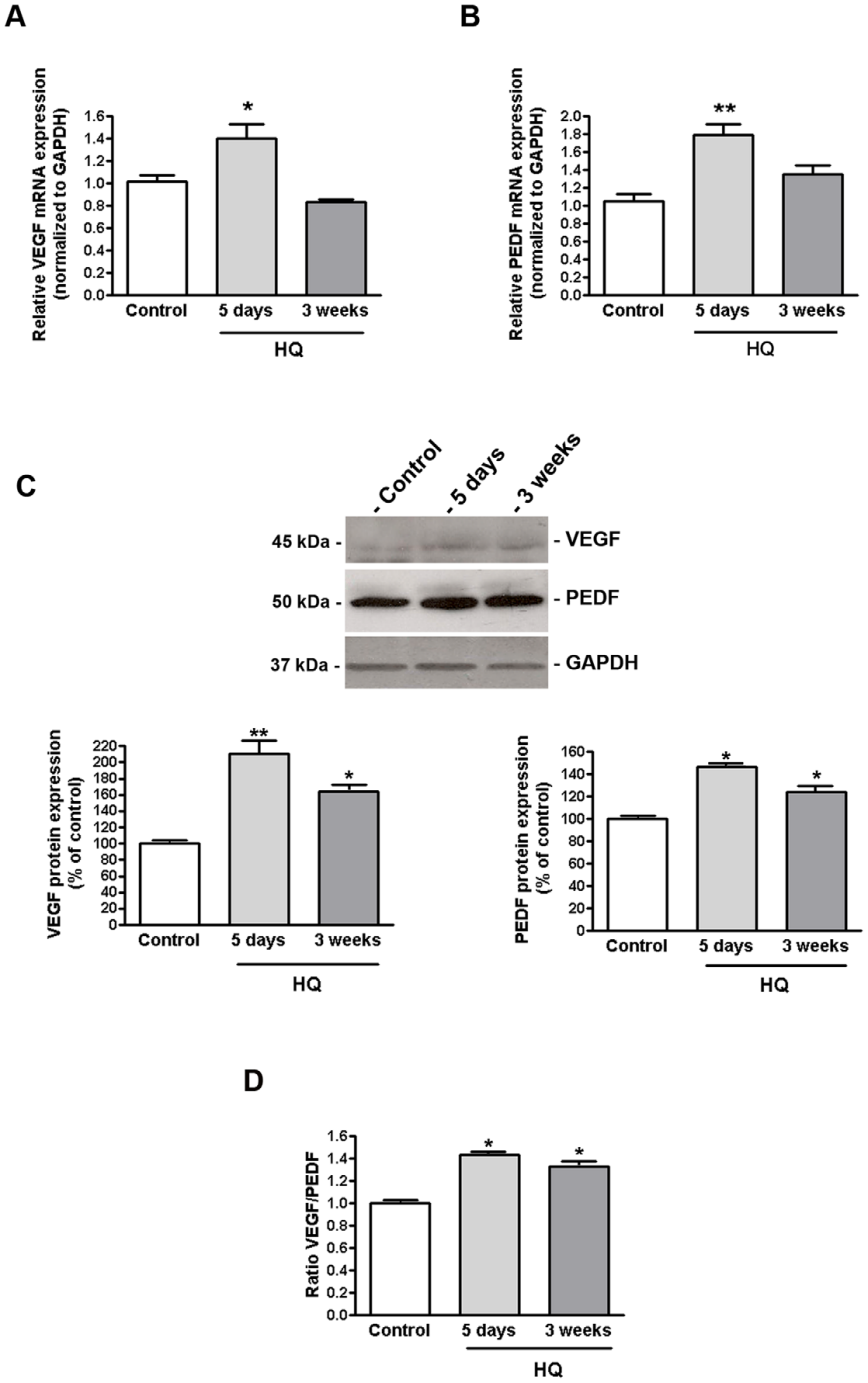
VEGF-PEDF balance is altered in RPE/choroids from mice exposed to HQ. (**A**) VEGF and (**B**) PEDF mRNA expression in response to HQ-induced oxidative injury. Total RNA was extracted from microdissected RPE/choroid complexes after 5 days and 3 weeks of exposure to HQ in drinking water (0.8%). VEGF and PEDF mRNA expression was measured by real-time PCR. GAPDH was used as internal control (n = 5 eyes per group). (**C**) VEGF and PEDF protein expression in response to HQ-induced oxidative injury. (D) VEGF-to-PEDF protein ratio. Total protein was extracted from microdissected RPE/choroid complexes after 5 days and 3 weeks of exposure to HQ in drinking water (0.8%). Equivalent amounts of protein from 5 eyes per group were pooled for each lane. VEGF and PEDF protein expression was evaluated by Western blot and normalized to GAPDH. *Top:* representative Western blots of the indicated proteins. The numbers to the left are molecular weights in kilodaltons (KDa). *Bottom*: average densitometry results. Data are expressed as percentage of control and are means ± SE. * is p<0.05 and **p<0.01 versus control.

The observations reported above led us to investigate the impact of HQ-induced oxidative damage on VEGF and PEDF expression in RPE/choroids from C57BL/6 mice treated with 0.8% HQ in drinking water for different periods of time. Our data show that VEGF mRNA levels were ∼40% higher than in control mice after exposure to HQ for 5 days (1.40±0.13 versus 1.02±0.05%, p<0.01) (Fig. 7A) whereas the expressed protein was upregulated by ∼109% (208.0±12.1 versus 100.0±0.02%, p<0.01) (Fig. 7C). However, VEGF mRNA expression was not modified after 3 consecutive weeks of oxidative stress (0.87±0.03 versus 1.02±0.05, p>0.05) (Fig. 7A) whereas VEGF protein expression was increased by ∼60% relative to control mice (160.3±6.4 versus 100.0±0.02%, p<0.05) (Fig. 7C). In addition, 5 days of exposure to HQ led to ∼80% increase in PEDF mRNA expression relative to control (1.80±0.11 versus 1.00±0.07%, p<0.01) (Fig. 7B) while the protein levels were increased by ∼50% (149.7±1.5 versus 100.0±0.03%, p<0.05) (Fig. 7C). After 3 weeks of oxidative stress with HQ, PEDF mRNA expression was not modified in RPE/choroids from HQ-treated mice (1.25±0.1 versus 1.00±0.07, p>0.05) (Fig. 7B) and PEDF protein expression was increased by ∼25% compared to control (124.8±0.06 versus 100.0±0.03%, p<0.05) (Fig. 7C). Overall, exposure to HQ for 5 days and 3 weeks enhanced the VEGF-to-PEDF ratio in mice RPE/choroid complexes by ∼45% (1.45±0.04 versus 1.0±0.01, p<0.05) and 30% (1.33±0.06 versus 1.0±0.01, p<0.05) respectively (Fig. 7D). Our data demonstrate changes in the levels of VEGF and PEDF in RPE/choroids from mice following HQ-induced oxidative injury revealing a disturbance of the angiogenic homeostatic balance which may play a role in the development of CNV.

## Discussion

Results of these experiments indicate that expression of MCP-1, VEGF and PEDF, key mediators pertinent to inflammation and angiogenesis are dysregulated in RPE from smoker patients with AMD as well as in cultured human RPE cells and RPE/choroids from C57BL/6 mice in response to various levels of pro-oxidant HQ and durations of exposure. These observations may have strong implications for the formation and accumulation of drusen and progression to CNV in smoker patients with dry AMD.

Although a cause and effect relationship has not been established, the presence, size and area covered by drusen within Bruch's membrane constitute a strong epidemiological risk indicator for the development and progression of the blinding end-stage form of AMD [8], [9], [10]. The mechanism of how drusen might elicit CNV remains undefined. However, evidence implicates cigarette smoking, oxidative stress and inflammation as important components in the pathogenesis and progression of the disease [23], [24], [25], [26], [27], [32]. While it is largely recognized that macrophages accumulate in AMD lesions [57], [58], [59], [60], [61], [62], [63], there is ambiguity surrounding their role in the disease process with conflicting evidence regarding whether they might be helpful by scavenging accumulated debris and therefore protecting against CNV or harmful by stimulating CNV [64]. This might be due to the largely observational nature of human samples but also probably reflects different functions macrophages serve during distinct phases of the disease. Previous observations made using mouse animal models suggest that MCP-1 and chemokine receptor–2 might be involved in the etiology of AMD and that macrophage dysfunction may have a central role in the early stage of AMD development [37], [38]. Indeed, it has been proposed that the spontaneous disappearance of drusen in patients with AMD may be explained by phagocytosis of debris thereby indicating a beneficial role of macrophages [31]. Although it has been recently demonstrated that previously described features of MCP-1 knockout ice that are similar to AMD [37] may only be age-related changes [65], the lack of direct studies on MCP-1 in human AMD prompted us to examine the expression of this major pro-inflammatory cytokine in patients diagnosed with the disease. Here, we report for the first time that MCP-1 expression is markedly decreased in RPE from smoker patients with AMD thereby pointing to a critical role for MCP-1 in the pathogenesis of the disease. We acknowledge that due to the nature of our study, it cannot be determined whether the altered expression of MCP-1 in human RPE lysates is a cause or consequence of the disease. However, our current findings suggest that declining MCP-1 production by aging RPE cells may impair recruitment of macrophages essential for scavenging debris which may lead to drusen formation and accumulation in AMD patients. Because cigarette smoking and oxidative stress have been unequivocally linked to the pathogenesis of AMD [66], it can be speculated that declining RPE-derived MCP-1 production resulting from cumulative exposure to oxidative damage may be an important factor that could accelerate and promote the progression of early AMD to CNV in smokers. This theory is put forth bearing in mind the complexity of underlying cellular and molecular mechanisms involved in the inflammatory response and with the acknowledgment that numerous AMD genotypes may exist. Therefore, we fully recognize that only some aspects of the proposed hypothesis may be involved in any given AMD genotype.

Based on the above hypothesis, we next evaluated the possibility that HQ-induced oxidative stress might regulate MCP-1 expression in the RPE. We showed that prolonged exposure to HQ-induced oxidative injury for 5 days and 3 weeks downregulated MCP-1 production by ARPE-19 cells and RPE/choroids from C57BL/6 mice. An earlier study by Joly et al is in line with our observations showing a decline in MCP-1 gene expression in retinas of mice exposed to light-induced oxidative damage for several days [67]. Our findings suggest that diminished infiltration of scavenging macrophages which might result from decreased MCP-1 production in response to HQ-induced oxidative injury to the RPE may lead to incomplete removal of cellular debris, owing overwhelming drusen accumulation which has been shown to be a risk factor for progression to CNV. Altogether, our findings highlight a particular significance for MCP-1 in the pathogenesis of AMD. Our observations suggest that sustained exposure to oxidative stress might impair RPE-derived MCP-1-mediated scavenging macrophages recruitment and phagocytosis which might lead to incomplete clearance of proinflammatory debris, infiltration of proangiogenic macrophages and ultimately to progressive drusen accumulation and CNV in smoker patients with AMD.

Inappropriate expression of VEGF and PEDF promoting functional overactivity of proangiogenic signaling has been associated to CNV [40]. We report here that VEGF expression is increased and PEDF expression is decreased in RPE from smoker patients with AMD resulting in an increased VEGF-to-PEDF ratio. A disruption in the critical balance of these opposing stimuli that may be permissive for the development of wet AMD. Our findings are consistent with clinical observations describing dysregulated expression of VEGF and PEDF [46], [47], [48], [53], [54], [56] in eyes with AMD. Oxidant-mediated RPE damage might promote abnormal angiogenesis [40]. Therefore, we next investigated whether HQ-induced oxidative stress might also regulate VEGF and PEDF expression in RPE cells. In vitro, although oxidative injury declined the production of PEDF without significantly changing VEGF expression in ARPE-19 cells regardless of dose and duration of exposure to HQ, we observed an increased VEGF-to-PEDF ratio which may favor angiogenesis. These results suggest that cigarette smoke-related HQ-induced oxidative stress might impair the delicate balance between VEGF and PEDF that controls angiogenic homeostasis in the retina. Other previous reports showed that cigarette smoke extract induces VEGF expression in ARPE-19 cells [23] and that H_2_O_2_-induced oxidative stress increased the production of VEGF in human RPE cells [40]. The discrepancy between our in vitro findings and those earlier observations with regards to VEGF might reflect differences in cellular responses in the setting of different types of oxidant-mediated injury. The endogenous angiogenic inhibitors are believed to be essential for maintaining the homeostasis of angiogenesis in the retina. Given the evidence that PEDF is an important negative regulator of angiogenesis, lower levels of PEDF is strongly suggestive of a decreased anti-angiogenic activity that may lead to the initiation of angiogenesis in response to HQ-induced oxidative stress. However, we do not rule out the possibility that a decreased level of inhibitory factor PEDF by itself may not be sufficient for inducing the angiogenic switch leading to CNV. Reciprocal increase in stimulatory VEGF might also be needed. In fact, a longer more sustained exposure to HQ might be necessary to induce VEGF expression in ARPE-19 cells. Furthermore, angiogenesis is a highly complex and tightly orchestrated multistep process involving extensive interplay between multiple angiogenic factors. It is therefore possible that several other molecules besides VEGF and PEDF regulated by HQ might permit the development of abnormal angiogenesis. In vivo, we observed elevated expression of VEGF and PEDF protein in RPE/choroids from HQ-treated mice which translated into an enhanced VEGF-to-PEDF ratio. As stated earlier, important species-specific differences may also account for the discrepancy between human cells and mice results. In addition, one has to keep in mind that inherent in vitro and in vivo differences might explain this disparity. PEDF has multiple dose-dependent biological functions. Interestingly, it has been reported that low doses of PEDF are inhibitory but high doses can increase the development of CNV induced by laser in mice [68]. In addition, a study showed that RPE-derived VEGF upregulates PEDF expression via VEGF receptor-1 in an autocrine manner [69], therefore highlighting regulatory interactions between these two counterbalancing systems of angiogenic stimulators and inhibitors. In any case, our in vivo findings confirm that HQ-induced oxidative damage is unequivocally associated with an imbalance between VEGF and PEDF in the RPE.

In summary, our data provide strong support for a key role played by injured RPE cells in the progression of dry AMD to CNV. We demonstrated that RPE dysfunction might lead to dysregulation of macrophage clearance function and angiogenic homeostasis as a result of oxidative damage which may trigger progression towards CNV in smoker patients with dry AMD.

## Materials and Methods

### Animal Ethics Statement

C57BL/6 mice were obtained from Jackson Laboratories (Bar Harbor, MN) and maintained at the McKnight Vision Research Center at University of Miami Miller School of Medicine in compliance with institutional regulations. University of Miami was granted full accreditation by the Association for Assessment and Accreditation of Laboratory Animal Care, International. This study was conducted according to the Association for Research in Vision and Ophthalmology statement for the Use of Animals in Ophthalmic and Vision Research and was approved under the University of Miami Care and Use Committee protocol number 10-050. The experiments were carried out in accordance with the Animal Welfare Act provisions and all other animal welfare guidelines for the Care and Use of Laboratory Animals.

### Human Ethics Statement

Human RPE lysates were prepared from cadaver eyes obtained from the Florida Lions Eye Bank determined unsuitable for transplantation (Miami, FL) (n = 4 patients diagnosed with AMD and n = 4 control non-smoker donors with no known history of eye disease). Methods for securing these eyes were humane, included proper written consent and approval and complied with the tenets of the Declaration of Helsinki. Written consent for the use of the eyes was obtained by the eye bank either through premortem instructions or from postmortem next of kin. The research was conducted with local University of Miami Institutional Review Boards committee approval (IRB # 19920231). Table 1 provides available information about the eye donors.

**Table 1:** Human Donor Eyes Information.

Samples		Control donors			AMD donors	
	Age	Gender	Smoker	Age	Gender	Smoker
1	64	M	No			
2	85	F	No			
3	69	M	No			
4	74	M	No			
5				70	M	Yes
6				68	M	Yes
7				81	M	?
8				75	M	Yes

**F**, female; **M**, male.

**?** Information on smoking status was unavailable.

### Cells and treatments

ARPE-19 cells, a human retinal pigment epithelial cell line [70] obtained from American Type Culture Collection (Manassas, VA) were grown to confluence in Dulbecco's Modified Eagles Medium/Ham's F-12 (DMEM/F12) (1∶1 vol/vol) medium supplemented with 10% fetal bovine serum (FBS), 1 mM L-glutamine, 100 µg/ml penicillin/streptomycin, and 0.348% Na_2_CO_3_ in a 5% CO_2_ humidified air incubator at 37°C. ARPE-19 cells are spontaneously immortalized human RPE cells that form polarized epithelial monolayers, are accepted as a model of RPE behavior and have been used in many studies. It has been demonstrated that ARPE-19 cells have structural and functional properties characteristic of RPE cells in vivo therefore suggesting that this cell line is valuable for in vitro studies of RPE physiology [70]. Cells were used between passage 5 and 8. For experiments, cells were split and plated at sub-confluent density in six-well and twenty four-well plates and grown to confluence. At the time of confluence, the medium was replaced for 48 hours with phenol red-free DMEM/F12 supplemented with 10% FBS medium. Subsequently, cells were treated following 2 protocols: Protocol 1 (sustained injury): HQ 10 µM every 24 hours for 5 days in phenol red-free DMEM/F12 supplemented with 1% FBS medium; Protocol 2 (repetitive injury): HQ 50 µM for 24 hours every 4 days for 3 weeks in phenol red-free DMEM/F12 supplemented with 0.1% FBS medium. The number of surviving cells was measured by cell count at the end of the treatment (Z1 Coulter Particle Counter, Beckman Coulter, Hialeah, FL) After treatment, supernatants were collected and cells were washed with phosphate buffered saline (PBS) and harvested in TRI Reagent (Sigma-Aldrich, St Louis, MO) for RNA extraction. All experiments (triplicate wells for each condition) were performed in triplicate wells at least in triplicate.

### Animals

Four-month-old male C57BL/6 mice were divided into 3groups: Group 1: control mice receiving regular drinking tap water (n = 5); Group 32: mice receiving HQ orally in drinking water (0.8% HQ) for 5 days (n = 5); Group 3: mice receiving HQ orally in drinking water (0.8% HQ) for 3 weeks (n = 5). Mice were housed in plastic cages with free access to food and water, and were kept on a 12-hour light–dark cycle. At the end of the experimental period, mice were euthanized with CO_2_ and eyes were immediately removed and microdissected for recovery of RPE/choroid sheets as previously described [71]. Total RNA and protein were extracted and stored at −80°C.

### Western blot

Western blot analysis was performed as previously described [29]. As information on smoking status as unavailable for one AMD donor, only 3 smoker patients with AMD and 3 non smoker controls were included in each group. Total protein was extracted in protein lysis buffer M-PER (Pierce, Rockford, IL) from human RPE and RPE/choroids isolated from mice and quantified by a Detergent Compatible protein assay (Bio-Rad, Hercules, CA). The primary antibodies used were as follows: anti-MCP-1 (dilution 1∶500, Cell Signaling, Danvers, MA), anti-VEGF (dilution 1∶500, LifeSpan, Seattle, WA) and anti-PEDF (dilution 1∶500, R&D Systems, Minneapolis, MN). Equal loading of gels was confirmed both by Ponceau S (Sigma-Aldrich, St Louis, MO) staining of membranes and GAPDH detection (Cell Signaling, Danvers, MA). The secondary antibodies used were horseradish-peroxidase-linked donkey anti-rabbit, anti-mouse or anti-goat (1∶1,000, Santa Cruz, Santa Cruz, CA).

### RNA extraction and real-time PCR

RNA extraction and real-time PCR were performed as previously described [29]. RNA was extracted in TRI reagent (Sigma-Aldrich, St Louis, MO) from ARPE-19 cells and RPE/choroids isolated from mice. Primer sequences and size of the product for each targeted gene are described in Table 2. Human and mouse GAPDH, MCP-1, VEGF and human PEDF primer sequences are available in the public RTPrimerDB database (http://medgen.UGent.be/rtprimerdb/) [72]. Mouse PEDF primer sequences were previously published [73]. Human Iκ-Bα and RelA/p65 primer sequences are available in the qPrimerDepot database (http://primerdepot.nci.nih.gov/).

**Table 2:** Real-time PCR Primers.

Target Gene	Forward Primer	Reverse Primer	Size (bp)
**Human**			
*GAPDH*	**tgc acc acc aac tgc tta gc**	**ggc atg gac tgt ggt cat gag**	**87**
*MCP-1*	**gtc tct gcc gcc ctt ctg t**	**ttg cat ctg gct gag cga g**	**76**
*VEGF*	**agg agg agg gca gaa tca tca**	**ctc gat tgg atg gca gta gct**	**76**
*PEDF*	**tcc aat gca gag gag tag ca**	**tgt gca ggc tta gag gga ct**	**93**
*RelA/p65*	**ggt ccg ctg aaa gga ctc tt**	**gaa ttc cag tac ctg cca ga**	**110**
*IkB-a*	**aaa gcc agg tct ccc ttc ac**	**cag cag ctc acc gag gac**	**107**
**Mouse**			
*GAPDH*	**cat ggc cct ccg tgt tcc ta**	**gcg gca cgt cag atc ca**	**55**
*MCP-1*	**agg tgt ccc aaa gaa get gta**	**atg tct gga ccc att cct tct**	**85**
*VEGF*	**gga gat cct tcg agg agc act t**	**ggc gat tta gca gca gat ata aga a**	**130**
*PEDF*	**tcg aaa gca gcc ctg tgt t**	**aat cac ccg act tca gca aga**	**N/A**

**N/A** = not available.

### ELISA

Cell supernatants were collected and protein concentration was determined by a Detergent Compatible protein assay. Concentrations of MCP-1, VEGF (R&D Systems, Minneapolis, MN) and PEDF (Millipore Corporation, Bedford, MA) were determined using ELISA kits following manufacturer's instructions. MCP-1, VEGF and PEDF levels were expressed relative to total protein (ng/mg of total protein) and subsequently expressed as % of control. All assays were done in duplicate.

### Statistics

Statistical analysis was performed with GraphPad Prism 4.0 (San Diego, CA). Data are expressed as percentage of the control and presented as means ± SEM of three to six independent experiments performed in triplicate. Statistical analysis of data was performed by using a one-way ANOVA followed by either a Dunnett or a Bonferroni multiple comparison post-hoc test, or the Student's t test. The level of significance accepted was p<0.05.

## References

[1] Augood CA, Vingerling JR, de Jong PT, Chakravarthy U, Seland J (2006). Prevalence of age-related maculopathy in older Europeans: the European Eye Study (EUREYE).. Arch Ophthalmol.

[2] Evans JR (2001). Risk factors for age-related macular degeneration.. Prog Retin Eye Res.

[3] Javitt JC, Zhou Z, Maguire MG, Fine SL, Willke RJ (2003). Incidence of exudative age-related macular degeneration among elderly Americans.. Ophthalmology.

[4] Klein R, Peto T, Bird A, Vannewkirk MR (2004). The epidemiology of age-related macular degeneration.. Am J Ophthalmol.

[5] Friedman DS, O'Colmain BJ, Munoz B, Tomany SC, McCarty C (2004). Prevalence of age-related macular degeneration in the United States.. Arch Ophthalmol.

[6] Rein DB, Wittenborn JS, Zhang X, Honeycutt AA, Lesesne SB (2009). Forecasting age-related macular degeneration through the year 2050: the potential impact of new treatments.. Arch Ophthalmol.

[7] Young RW (1987). Pathophysiology of age-related macular degeneration.. Surv Ophthalmol.

[8] Bressler SB, Maguire MG, Bressler NM, Fine SL (1990). Relationship of drusen and abnormalities of the retinal pigment epithelium to the prognosis of neovascular macular degeneration. The Macular Photocoagulation Study Group.. Arch Ophthalmol.

[9] Sarks SH, Van Driel D, Maxwell L, Killingsworth M (1980). Softening of drusen and subretinal neovascularization.. Trans Ophthalmol Soc U K.

[10] Vinding T (1990). Occurrence of drusen, pigmentary changes and exudative changes in the macula with reference to age-related macular degeneration. An epidemiological study of 1000 aged individuals.. Acta Ophthalmol (Copenh).

[11] Swaroop A, Branham KE, Chen W, Abecasis G (2001). Genetic susceptibility to age-related macular degeneration: a paradigm for dissecting complex disease traits.. Hum Mol Genet.

[12] Smith W, Assink J, Klein R, Mitchell P, Klaver CC (2001). Risk factors for age-related macular degeneration: Pooled findings from three continents.. Ophthalmology.

[13] Christen WG, Glynn RJ, Manson JE, Ajani UA, Buring JE (1996). A prospective study of cigarette smoking and risk of age-related macular degeneration in men.. JAMA.

[14] Seddon JM, Willett WC, Speizer FE, Hankinson SE (1996). A prospective study of cigarette smoking and age-related macular degeneration in women.. JAMA.

[15] Dhubhghaill SS, Cahill MT, Campbell M, Cassidy L, Humphries MM (2010). The pathophysiology of cigarette smoking and age-related macular degeneration.. Adv Exp Med Biol.

[16] Smith W, Mitchell P, Leeder SR (1996). Smoking and age-related maculopathy. The Blue Mountains Eye Study.. Arch Ophthalmol.

[17] Klein R, Klein BE, Linton KL, DeMets DL (1993). The Beaver Dam Eye Study: the relation of age-related maculopathy to smoking.. Am J Epidemiol.

[18] Tamakoshi A, Yuzawa M, Matsui M, Uyama M, Fujiwara NK (1997). Smoking and neovascular form of age related macular degeneration in late middle aged males: findings from a case-control study in Japan. Research Committee on Chorioretinal Degenerations.. Br J Ophthalmol.

[19] Delcourt C, Diaz JL, Ponton-Sanchez A, Papoz (1998). Smoking and age-related macular degeneration. The POLA Study. Pathologies Oculaires Liees a l'Age.. Arch Ophthalmol.

[20] Thornton J, Edwards R, Mitchell P, Harrison RA, Buchan I (2005). Smoking and age-related macular degeneration: a review of association.. Eye (Lond).

[21] Klein R, Knudtson MD, Cruickshanks KJ, Klein BE (2008). Further observations on the association between smoking and the long-term incidence and progression of age-related macular degeneration: the Beaver Dam Eye Study.. Arch Ophthalmol.

[22] Khan JC, Thurlby DA, Shahid H, Clayton DG, Yates JR (2006). Smoking and age related macular degeneration: the number of pack years of cigarette smoking is a major determinant of risk for both geographic atrophy and choroidal neovascularisation.. Br J Ophthalmol.

[23] Bertram KM, Baglole CJ, Phipps RP, Libby RT (2009). Molecular regulation of cigarette smoke induced-oxidative stress in human retinal pigment epithelial cells: implications for age-related macular degeneration.. Am J Physiol Cell Physiol.

[24] Wang AL, Lukas TJ, Yuan M, Du N, Handa JT (2009). Changes in retinal pigment epithelium related to cigarette smoke: possible relevance to smoking as a risk factor for age-related macular degeneration.. PLoS One.

[25] Espinosa-Heidmann DG, Suner IJ, Catanuto P, Hernandez EP, Marin-Castano ME (2006). Cigarette smoke-related oxidants and the development of sub-RPE deposits in an experimental animal model of dry AMD.. Invest Ophthalmol Vis Sci.

[26] Marin-Castano ME, Striker GE, Alcazar O, Catanuto P, Espinosa-Heidmann DG (2006). Repetitive nonlethal oxidant injury to retinal pigment epithelium decreased extracellular matrix turnover in vitro and induced sub-RPE deposits in vivo.. Invest Ophthalmol Vis Sci.

[27] Marin-Castano ME, Csaky KG, Cousins SW (2005). Nonlethal oxidant injury to human retinal pigment epithelium cells causes cell membrane blebbing but decreased MMP-2 activity.. Invest Ophthalmol Vis Sci.

[28] Strunnikova N, Zhang C, Teichberg D, Cousins SW, Baffi J (2004). Survival of retinal pigment epithelium after exposure to prolonged oxidative injury: a detailed gene expression and cellular analysis.. Invest Ophthalmol Vis Sci.

[29] Pons M, Cousins SW, Csaky KG, Striker G, Marin-Castano ME (2010). Cigarette Smoke-Related Hydroquinone Induces Filamentous Actin Reorganization and Heat Shock Protein 27 Phosphorylation through p38 and Extracellular Signal-Regulated Kinase 1/2 in Retinal Pigment Epithelium. Implications for Age-Related Macular Degeneration.. Am J Pathol.

[30] Patel M, Chan CC (2008). Immunopathological aspects of age-related macular degeneration.. Semin Immunopathol.

[31] Hageman GS, Luthert PJ, Victor Chong NH, Johnson LV, Anderson DH (2001). An integrated hypothesis that considers drusen as biomarkers of immune-mediated processes at the RPE-Bruch's membrane interface in aging and age-related macular degeneration.. Prog Retin Eye Res.

[32] Donoso LA, Kim D, Frost A, Callahan A, Hageman G (2006). The role of inflammation in the pathogenesis of age-related macular degeneration.. Surv Ophthalmol.

[33] Haines JL, Hauser MA, Schmidt S, Scott WK, Olson LM (2005). Complement factor H variant increases the risk of age-related macular degeneration.. Science.

[34] Lommatzsch A, Hermans P, Muller KD, Bornfeld N, Bird AC (2008). Are low inflammatory reactions involved in exudative age-related macular degeneration? Morphological and immunhistochemical analysis of AMD associated with basal deposits.. Graefes Arch Clin Exp Ophthalmol.

[35] Uetama T, Ohno-Matsui K, Nakahama K, Morita I, Mochizuki M (2003). Phenotypic change regulates monocyte chemoattractant protein-1 (MCP-1) gene expression in human retinal pigment epithelial cells.. J Cell Physiol.

[36] Higgins GT, Wang JH, Dockery P, Cleary PE, Redmond HP (2003). Induction of angiogenic cytokine expression in cultured RPE by ingestion of oxidized photoreceptor outer segments.. Invest Ophthalmol Vis Sci.

[37] Ambati J, Anand A, Fernandez S, Sakurai E, Lynn BC (2003). An animal model of age-related macular degeneration in senescent Ccl-2- or Ccr-2-deficient mice.. Nat Med.

[38] Tuo J, Bojanowski CM, Zhou M, Shen D, Ross RJ (2007). Murine ccl2/cx3cr1 deficiency results in retinal lesions mimicking human age-related macular degeneration.. Invest Ophthalmol Vis Sci.

[39] Holtkamp GM, De Vos AF, Peek R, Kijlsta A (1999). Analysis of the secretion pattern of monocyte chemotactic protein-1 (MCP-1) and transforming growth factor-beta 2 (TGF-beta2) by human retinal pigment epithelial cells.. Clin Exp Immunol.

[40] Ohno-Matsui K, Morita I, Tombran-Tink J, Mrazek D, Onodera M (2001). Novel mechanism for age-related macular degeneration: an equilibrium shift between the angiogenesis factors VEGF and PEDF.. J Cell Physiol.

[41] Adamis AP, Shima DT, Yeo KT, Yeo TK, Brown LF (1993). Synthesis and secretion of vascular permeability factor/vascular endothelial growth factor by human retinal pigment epithelial cells.. Biochem Biophys Res Commun.

[42] Husain D, Ambati B, Adamis AP, Miller JW (2002). Mechanisms of age-related macular degeneration.. Ophthalmol Clin North Am.

[43] Kwak N, Okamoto N, Wood JM, Campochiaro PA (2000). VEGF is major stimulator in model of choroidal neovascularization.. Invest Ophthalmol Vis Sci.

[44] Tong JP, Yao YF (2006). Contribution of VEGF and PEDF to choroidal angiogenesis: a need for balanced expressions.. Clin Biochem.

[45] Witmer AN, Vrensen GF, Van Noorden CJ, Schlingemann RO (2003). Vascular endothelial growth factors and angiogenesis in eye disease.. Prog Retin Eye Res.

[46] Kliffen M, Sharma HS, Mooy CM, Kerkvliet S, de Jong PT (1997). Increased expression of angiogenic growth factors in age-related maculopathy.. Br J Ophthalmol.

[47] Kvanta A, Algvere PV, Berglin L, Seregard S (1996). Subfoveal fibrovascular membranes in age-related macular degeneration express vascular endothelial growth factor.. Invest Ophthalmol Vis Sci.

[48] Lopez PF, Sippy BD, Lambert HM, Thach AB, Hinton DR (1996). Transdifferentiated retinal pigment epithelial cells are immunoreactive for vascular endothelial growth factor in surgically excised age-related macular degeneration-related choroidal neovascular membranes.. Invest Ophthalmol Vis Sci.

[49] Reich SJ, Fosnot J, Kuroki A, Tang W, Yang X (2003). Small interfering RNA (siRNA) targeting VEGF effectively inhibits ocular neovascularization in a mouse model.. Mol Vis.

[50] Olsson AK, Dimberg A, Kreuger J, Claesson-Welsh L (2006). VEGF receptor signalling - in control of vascular function.. Nat Rev Mol Cell Biol.

[51] Blaauwgeers HG, Holtkamp GM, Rutten H, Witmer AN, Koolwijk P (1999). Polarized vascular endothelial growth factor secretion by human retinal pigment epithelium and localization of vascular endothelial growth factor receptors on the inner choriocapillaris. Evidence for a trophic paracrine relation.. Am J Pathol.

[52] Yi X, Ogata N, Komada M, Yamamoto C, Takahashi K (1997). Vascular endothelial growth factor expression in choroidal neovascularization in rats.. Graefes Arch Clin Exp Ophthalmol.

[53] Dawson DW, Volpert OV, Gillis P, Crawford SE, Xu H (1999). Pigment epithelium-derived factor: a potent inhibitor of angiogenesis.. Science.

[54] Bhutto IA, McLeod DS, Hasegawa T, Kim SY, Merges C (2006). Pigment epithelium-derived factor (PEDF) and vascular endothelial growth factor (VEGF) in aged human choroid and eyes with age-related macular degeneration.. Exp Eye Res.

[55] Ogata N, Wada M, Otsuji T, Jo N, Tombran-Tink J (2002). Expression of pigment epithelium-derived factor in normal adult rat eye and experimental choroidal neovascularization.. Invest Ophthalmol Vis Sci.

[56] Holekamp NM, Bouck N, Volpert O (2002). Pigment epithelium-derived factor is deficient in the vitreous of patients with choroidal neovascularization due to age-related macular degeneration.. Am J Ophthalmol.

[57] Penfold PL, Madigan MC, Gillies MC, Provis JM (2001). Immunological and aetiological aspects of macular degeneration.. Prog Retin Eye Res.

[58] Killingsworth MC, Sarks JP, Sarks SH (1990). Macrophages related to Bruch's membrane in age-related macular degeneration.. Eye (Lond).

[59] Dastgheib K, Green WR (1994). Granulomatous reaction to Bruch's membrane in age-related macular degeneration.. Arch Ophthalmol.

[60] Grossniklaus HE, Ling JX, Wallace TM, Dithmar S, Lawson DH (2002). Macrophage and retinal pigment epithelium expression of angiogenic cytokines in choroidal neovascularization.. Mol Vis.

[61] Lopez PF, Grossniklaus HE, Lambert HM, Aaberg TM, Capone A (1991). Pathologic features of surgically excised subretinal neovascular membranes in age-related macular degeneration.. Am J Ophthalmol.

[62] Oh H, Takagi H, Takagi C, Suzuma K, Otani A (1999). The potential angiogenic role of macrophages in the formation of choroidal neovascular membranes.. Invest Ophthalmol Vis Sci.

[63] Yi X, Takahashi K, Ogata N, Uyama M (1996). Immunohistochemical proof of origin of macrophages in laser photocoagulation lesion in the retina.. Jpn J Ophthalmol.

[64] Ding X, Patel M, Chan CC (2009). Molecular pathology of age-related macular degeneration.. Prog Retin Eye Res.

[65] Luhmann UF, Robbie S, Munro PM, Barker SE, Duran Y (2009). The drusenlike phenotype in aging Ccl2-knockout mice is caused by an accelerated accumulation of swollen autofluorescent subretinal macrophages.. Invest Ophthalmol Vis Sci.

[66] Beatty S, Koh H, Phil M, Henson D, Boulton M (2000). The role of oxidative stress in the pathogenesis of age-related macular degeneration.. Surv Ophthalmol.

[67] Joly S, Samardzija M, Wenzel A, Thiersch M, Grimm C (2009). Nonessential role of beta3 and beta5 integrin subunits for efficient clearance of cellular debris after light-induced photoreceptor degeneration.. Invest Ophthalmol Vis Sci.

[68] Apte RS, Barreiro RA, Duh E, Volpert O, Ferguson TA (2004). Stimulation of neovascularization by the anti-angiogenic factor PEDF.. Invest Ophthalmol Vis Sci.

[69] Ohno-Matsui K, Yoshida T, Uetama T, Mochizuki M, Morita I (2003). Vascular endothelial growth factor upregulates pigment epithelium-derived factor expression via VEGFR-1 in human retinal pigment epithelial cells.. Biochem Biophys Res Commun.

[70] Dunn KC, Aotaki-Keen AE, Putkey FR, Hjelmeland LM (1996). ARPE-19, a human retinal pigment epithelial cell line with differentiated properties.. Exp Eye Res.

[71] Praddaude F, Cousins SW, Pecher C, Marin-Castano ME (2009). Angiotensin II-induced hypertension regulates AT1 receptor subtypes and extracellular matrix turnover in mouse retinal pigment epithelium.. Exp Eye Res.

[72] Vandesompele J, De Preter K, Pattyn F, Poppe B, Van Roy N (2002). Accurate normalization of real-time quantitative RT-PCR data by geometric averaging of multiple internal control genes.. Genome Biol.

[73] Yang H, Xu Z, Iuvone PM, Grossniklaus HE (2006). Angiostatin decreases cell migration and vascular endothelium growth factor (VEGF) to pigment epithelium derived factor (PEDF) RNA ratio in vitro and in a murine ocular melanoma model.. Mol Vis.

